# Navigating conflicting laws in sexual and reproductive health service provision for teenagers

**DOI:** 10.4102/curationis.v39i1.1565

**Published:** 2016-02-25

**Authors:** Kelley Moult, Alexandra Müller

**Affiliations:** 1 1University of Cape Town, Gender, Health and Justice Research Unit, Faculty of Health Sciences, South Africa

## Abstract

**Background:**

The South African legal and policy framework for sexual and reproductive healthcare provision for teenagers is complex.

**Objective:**

The article outlines the dilemmas emanating from the legal and policy framework, summarises issues with implementation of the legal and policy framework in practice, and summarises recent changes to the law.

**Methods:**

In-depth analysis of the legal and policy framework. Training workshops with a purposive sample of nurses and other healthcare providers in the Western Cape.

**Findings:**

Tensions between consent and confidentiality imposed by the *Termination of Pregnancy Act*, the *Children’s Act*, the *National Health Act* and the *Criminal Law* (Sexual Offences and Related Matters) *Amendment Act* render conflicting obligations on healthcare providers. Healthcare providers’ experiences with service provision in this context show that the conflicting roles they inhabit render their service provision to teenagers more challenging.

**Conclusion:**

Healthcare providers need to learn about their legal obligations surrounding adolescent sexual and reproductive health services.

## Introduction

In October 2013, the Constitutional Court delivered judgement in the so-called Teddy Bear Clinic Case, which challenged the constitutionality of provisions of the *Sexual Offences Act* (Criminal Law [Sexual Offences and Related Matters] *Amendment Act* 32 of 2007) relating to adolescents. The provisions in question directly implicated sexual and reproductive health (SRH) care providers because they criminalised a very wide range of consensual sexual activity between adolescents aged 12–15 years, including kissing on the mouth, hugging, sexual touching and sexual intercourse. These provisions also created mandatory requirements for ‘anyone’ with knowledge of consensual sexual activity to report this to the police, who were required to refer the case to the National Prosecuting Authority for a decision on how to proceed. Because the group of mandatory reporters is so widely defined, SRH care providers fall within this ambit. This means that, when faced with a teenager who wants to access contraception or other SRH services, healthcare providers are faced with a tricky choice between providing services and reporting the teen.

The intent of these provisions in the law was to protect teens from unwanted or ill-advised sexual activity, but in practice their implementation was much more problematic (illustrated, e.g. by the much-publicised Jules High School case that saw three teenagers prosecuted for consensual sexual activity). The crux of the Teddy Bear Clinic’s challenge to these sections of the law was that these provisions harmed the very adolescents they intended to protect. This argument was based on the fact that the sexual activity in question is developmentally age-appropriate and that criminalising such behaviour bars access to information for teenagers, unnecessarily exposes them to the criminal justice system, and potentially damages teenagers’ understanding of sexuality, as well as their opportunities to develop a healthy attitude towards their body and sexuality.

The review article provides an overview of the South African legislative framework that governed the provision of SRH services for adolescents between the age of 12 and 15 years (until July 2015) and highlights the apparent conflicts amongst these laws and policies. It analyses the dilemmas for healthcare providers, summarises the implications of the Constitutional Court judgement for providers and teenage patients and sets out the changes to the law brought about in July 2015 by the *Criminal Law Sexual Offences and Related Matters Amendment Act* 5 of 2015 (hereafter referred to as the *SOA Amendment Act*). Lastly, it presents strategies to provide healthcare providers with guidance when providing SRH services to adolescents. The article presents data from a qualitative study undertaken by the Gender, Health and Justice Research Unit of the University of Cape Town between 2012 and 2014 to understand healthcare workers’ experiences in understanding and implementing the legal framework on adolescent SRH care.

## The legal framework: Conflicting laws and policies

The provisions that were challenged in the *Teddy Bear Clinic Case* are part of the regulatory framework that shapes a particularly tricky aspect of SRH care service provision: services for adolescents. South Africa’s 1996 Constitution and Bill of Rights protect the right to make decisions regarding reproduction and the right to access healthcare services for both adults and children. Several laws breathe life into these constitutional rights and make them accessible to children. The *Choice on Termination of Pregnancy Act* 92 of 1996 (Republic of South Africa 1996) allows that women and girls of any age can request a termination of pregnancy (TOP) up to 12 weeks, and the *National Health Act* 61 of 2003 (Republic of South Africa 2003) mandates that all information concerning a patient (of any age) is confidential. The *Children’s Act* 38 of 2005 (Republic of South Africa 2005) states that that children from the age of 12 may not be refused condoms and contraceptives and that such provision must be kept confidential. All these laws, as well as the *Sexual Offences Act* 32 of 2007 (Republic of South Africa 2007), which says that children may only freely consent to sex at 16 years of age, regulate aspects of teenagers’ access to health care and sexual and reproductive rights and also specify obligations and responsibilities of healthcare workers who provide SRH services to teenagers. We discuss each act in detail below.

### The *Choice on Termination of Pregnancy Act*

One of the first pieces of legislation passed under the post-apartheid government, the *Choice on Termination of Pregnancy Act* 92 of 1996 (the CTPA), allows that any pregnant woman or girl can request a TOP up to 12 weeks of gestation (for more information, see note 1 in the clarification of terms at the end of the article), without consultation or approval by a doctor or nurse. The Act explicitly states that it applies to ‘any female person of any age’, and the courts have only limited this by adding the requirement that a child seeking an abortion also be able to provide informed consent. Minors are therefore not required to consult their parents before having an abortion (although healthcare providers should advise them to do so), and healthcare facilities may not deny children the service should they choose not to do so. Although preabortion (and post abortion) counselling is available to patients, they are not required to undergo counselling in order to access an abortion.

Structuring access in this way is intended to remove barriers to seeking help – for example, where pregnant minors may have been sexually abused by their father or guardian or where they are simply too afraid to speak to their parents about the issue. The law is very clear that abortions in the first trimester are the autonomous decision of the pregnant woman or girl and are not subject to any conditions or requirements other than the pregnant woman’s or girl’s informed consent. The CTPA also stipulates that the identity of a woman who has requested or obtained an abortion shall remain confidential at all times unless she chooses to disclose that information herself. Facilities that provide terminations of pregnancies are only required to keep records of the number of abortions they perform and to forward this information to the National Department of Health on a monthly basis.

### The *National Health Act*

The *National Health Act* 61 of 2003 (the NHA) deals with a wide range of health-related issues and is relevant in the context of SRH service provision because it addresses healthcare providers’ duties and patients’ rights. The NHA recognises that an informed decision about a medical procedure can only be made if the patient has been given all relevant information on the procedure’s benefits and potential risks. The Act therefore upgraded the ethical principle of patient confidentiality to a binding requirement for the provision of healthcare services (for more info, see note 2 in clarifications).

Patient confidentiality and informed consent are key principles of medicine – they underpin a trusting relationship between provider and patient and help ensure that patients feel comfortable in accessing preventative or curative health services and health information. Breaches in confidentiality are problematic because they prevent patients from accessing care, which not only negatively affects the patient’s own health but may also put others at risk – especially in a context of communicable and stigmatised health issues such as HIV and/or AIDS or (other) SRH concerns. Confidentiality and trust are critical issues in SRH services for teens, as this group are in need of credible information, but are often nervous about disclosing sexual activity or using available SRH services.

### The *Children’s Act*

The *Children’s Act* 38 of 2005 (which was finally promulgated in its entirety, along with the accompanying Regulations, in 2010) introduced a number of critically important principles in respect of *children. The Act* introduced the so-called ‘best interests of the child’ principle, which states that ‘a child’s best interests are of paramount importance in every matter concerning the child’, and that this standard must be applied in all matters concerning the care, protection and well-being of a child (see clarification 3). *The Act also* dropped the age of consent for most health-related decisions to 12 years in an effort to strengthen the autonomy of children in making decisions that affect them (including about their health). In terms of SRH services, the Act has a strong public health framing that views access to contraceptives (see clarification 4) as being in the best interests of children by allowing sexually active teenagers to protect themselves from unprotected sex and sexually transmitted diseases, including HIV. The right to contraception, however, is intended to go hand in hand with appropriate sexuality education. The Act views healthcare providers as being well placed, and adequately trained, to detect these needs and to provide the requisite care and education.

The *Children’s Act* also protects children’s rights to confidentiality about the provision of contraceptive services, as well as in terms of their health status more generally. This confidentiality is limited, however, by requiring certain professionals (amongst them healthcare providers) to report cases where they reasonably believe a child is a victim of abuse (see clarification 5) to either the provincial Department of Social Development, a designated child protection organisation, or a police officer, in order for the matter to be investigated and, where necessary, for the appropriate measures to be taken to protect the child from further harm. Failure to report the abuse of a child carries a penalty of a fine and/or imprisonment up to a maximum of 10 years.

### The *Criminal Law* (Sexual Offences & Related Matters) *Amendment Act*

The *Criminal Law* (Sexual Offences & Related Matters) *Amendment Act* 32 of 2007 (the SOA) redefined sexual offences under South African law – changing the definition of rape by making it gender neutral and widening the ambit of what constitutes rape and also introducing a range of other statutory offences that address nonconsensual sexual acts with adults and children.

The SOA set the age of consent to sex at 16 years – consensual sexual activity with people over 16 years old is therefore not a crime. The Act also mandated that children under the age of 12 are not able to give legally valid consent to sexual acts. In terms of the SOA, any sexual acts with a child under the age of 12 years were therefore regarded as nonconsensual (even where the child apparently consented) and were considered serious offences. (The SOA was amended in July 2015 by the *SOA Amendment Act*, which is discussed below.)

The law is applicable to children over 12, but the law for children younger than 16 is much more complex. The 2007 SOA created a special category of offences related to consensual sexual activity with children over 12 years, but under the age of 16. Nonconsensual sexual activity with anyone, of any age, is always a crime. The clauses in question – Sections 15 and 16 of the 2007 SOA – listed the offences of statutory rape (for penetrative acts) and statutory sexual assault (for nonpenetrative acts), even where both parties were children in this age group (see clarification 6). For these children, even though they consented to the sexual activity, that consent was not legally valid because of their age. These clauses covered a wide range of behaviours including direct or indirect contact between the mouth of one person and the genital organs, anus, female breasts or mouth of another person. Many of these behaviours are considered developmentally appropriate for children in this age group, for example, kissing, sexual touching and penetration with a finger or sex toy (WHO 2012; Monasterio *et al*. 2010). These complexities are illustrated in Table 1.

**TABLE 1:** Sexual activity with teenagers and children and interpretation under the Criminal Law (Sexual Offences and Related Matters) *Amendment Act* 32 of 2007 and *Criminal Law* (Sexual Offences and Related Matters) *Amendment Act* 5 of 2015, respectively.

Type	2007 *Sexual Offences Act*	2015 *Sexual Offences Amendment Act*	Offence
Nonconsensual sexual acts	Nonconsensual penetrative sex with a child under the age of 16 years	No change	Rape
	Nonconsensual nonpenetrative sex with any child under the age of 16 years	No change	Sexual assault
Consensual sexual activity	Consensual penetrative sex with a child between 12 and 15 years by an adult or another child	Consensual penetrative sex with a child between 12 and 15 years by an adult or another child aged 16-17 where the age difference between them is greater than 2 years	Statutory rape
	Consensual penetrative sex with a child under the age of 12	No change	Rape
	Consensual nonpenetrative sex with a child between 12 and 15 years by an adult or another child	Consensual nonpenetrative sex with a child between 12 and 15 years by an adult or another child aged 16-17 where the age difference between them is greater than 2 years	Statutory sexual assault
	Consensual nonpenetrative sex with a child under the age of 12		Sexual assault

In addition, Section 54(1)(a) of the 2007 *Sexual Offences Act* obligates *anyone* who has knowledge of the commission of a sexual offence against a child to report it to the South African Police Services immediately (see clarification 7). Section 54(1)(b) further places criminal sanctions for anyone who fails to report these offences. In fact, under the law a person who fails to report knowledge of a sexual offence can be liable to a fine or to imprisonment for up to 5 years, or both. Importantly, the reporting obligation under the SOA is different from the reporting obligation under the *Children’s Act*, which gives mandatory reporters (including healthcare providers) a range of places to report sexual abuse of a child, amongst them social workers or other designated child protection organisations. Under the SOA, knowledge of a sexual offence has to be reported to the police.

Whilst the purpose of these clauses in the 2007 SOA were to encourage the protection of children by placing everyone under an obligation to report abuse, the broad application of these provisions were widely viewed as encouraging interference with the privacy of children for reasons other than those that could be construed as being in the child’s best interests (McQuoid-Mason 2011).

### Providing healthcare services – balancing consent and confidentiality

The various provisions described above create an inherent tension between consent and confidentiality. In practice, these conflicting laws mean that SRH care providers, when seeing a teenager who wants to access contraception or other SRH services, are faced with a tricky choice between providing services, support and counselling for the teenager about their choices and reporting the teenager to police and social workers in order to enforce certain aspects of the law. These tensions between consent and confidentiality are summarised in Table 2.

**TABLE 2:** Confidentiality provisions in law.

Confidentiality provision	Law
A child’s right to confidentiality in respect of sexual reproductive health services is limited where a medical practitioner reasonably believes that the child has been abused or neglected.	*Children’s Act*
A child has the right to confidentiality in respect of information concerning her health status, treatment or stay in a health establishment except where records need to be disclosed in the best interest of the child, for a legitimate purpose or in the scope of a health practitioner’s duties.	*National Health Act*
A child does not have the right to confidentiality in respect of sexual activity as healthcare professionals are obligated to report knowledge of a sexual offence.	*Sexual Offences Act*

In the healthcare setting, much of the confusion that ensues from this complex legal framework relates to consent – at which age a teenager is able to consent to either sex and/or SRH services. Although a child only legally becomes an adult in law at the age of 18 years, the SOA states that children can legally consent to having sex from the age of 16. Children are entitled to SRH services such as contraceptives and HIV testing from the age of 12 (or younger) to encourage access to professional advice and health services. Because it is difficult to prevent teenagers from having sex, the law ensures that they can at least access preventive health services that protect them from sexually transmitted infections and teenage pregnancy. Table 3 illustrates the ages of consent to services.

**TABLE 3:** Age of consent to services.

Issue	Age of consent	Law
General medical treatment	Children can consent to medical treatment without the consent of a parent when they are over the age of 12, and have sufficient maturity and the mental capacity to understand the benefits, risks and social implications.	*Children’s Act*
HIV test	Children aged 12 years and above can consent to an HIV test without their parents’ consent. Children under 12 can consent to an HIV test without their parents’ consent if they have sufficient maturity and the mental capacity to understand the benefits, risks and social implications.	*Children’s Act*
Condoms	Children over the age of 12 years may not be refused condoms by a healthcare provider or condom seller.	*Children’s Act*
Contraceptives	Contraceptives other than condoms may be provided to a child aged 12 years and above without their parents’ consent. A medical examination must be done and proper advice given to the child.	*Children’s Act*
Sex	A person may legally consent to sexual activity (penetrative or nonpenetrative) at 16 years of age. (Note that this has not changed under the 2015 SOA Amendment Act).	*Sexual Offences Act* (2007)
Termination of pregnancy	Any pregnant woman or girl can request a termination of pregnancy up to 12 weeks of gestation, without consultation or approval by a doctor or nurse. This means that there is no age restriction for a TOP, and girls can consent without their parents. This is to ensure that any woman or girl who needs this service can access a termination of pregnancy.	*Termination of Pregnancy Act*

SOA, Sexual Offences Act; TOP, termination of pregnancy.

### The *Teddy Bear Clinic* case and law reform

In October 2013, the Constitutional Court delivered judgement in the case of the *Teddy Bear Clinic for Abused Children and Another v Minister of Justice and Constitutional Development and Another* (CCT 12/13) [2013] ZACC 35) – commonly known as the ‘Teddy Bear Clinic’ case. The applicants challenged the constitutionality of Sections 15 and 16 of the SOA, arguing that whilst these provisions intended to protect teenagers from unwanted or ill-advised sexual activity, their implementation has been highly problematic and has not always resulted in the ‘best interest of the child’ being upheld.

The applicants argued that the provisions of the SOA actually harmed adolescents, because consensual sexual activity, as outlined under the two sections, is appropriate for the levels of development of adolescents. The applicants further argued that the provisions were particularly punitive for girls in that if consensual sex resulted in pregnancy the healthcare provider who provided the girl with prenatal care or an abortion would be required to report the girl to the police and charges may result.

The applicants argued that Sections 15 and 16 were unconstitutional because they infringed on children’s rights to dignity, privacy, bodily and psychological integrity. Additionally, the applicants argued that the sections infringed on children’s right to have their best interest treated as being of paramount importance in all matters concerning them. Therefore, the court had to decide if it was constitutional that children faced criminal sanctions for developmentally appropriate, consensual sexual behaviour in order to delay sexual activity and reduce the risks associated with it.

The Constitutional Court agreed with the applicants’ argument and declared the provisions unconstitutional, because they imposed criminal liability on children under the age of 16 and contravened the ‘best interest of the child’ principle. The court gave the legislature 18 months to amend the SOA and criminal prosecutions against children under this law were stopped whilst the Parliament made changes to the law. The Department of Justice and Correctional Services offered opportunities for public submission and public comment during 2014 and early 2015 on the proposed amendments to the Bill. Numerous child-focused nongovernmental organisations advocated that the legislature should place the regulation of teenage sexual activity within the purview of health, social welfare and education rather than criminal justice. These organisations also advocated for the use of the ‘best interests of the child’ standard in amending the SOA to clarify children’s rights to privacy and confidentiality for service providers and to simplify the legal framework on SRH service provision for teens.

### The *Criminal Law* (Sexual Offences & Related Matters) *Amendment Act* 5 of 2015

The law reform process instituted as a result of the Teddy Bear Clinic Case culminated in July 2015 with the promulgation of the *Criminal Law* [Sexual Offences and Related Matters] *Amendment Act* 5 of 2015 (hereafter the 2015 *SOA Amendment Act*). The 2015 *SOA Amendment Act* made two changes to Sections 15 and 16. Firstly, it decriminalised consensual sexual activity between teenagers who are both between the ages of 12 and 15 years (regardless of the age difference between them). Secondly, it decriminalised consensual sexual activity where one teenager is between 12 and 15 years old, and the other between 16 and 17 years old *as long as the age difference between them is less than 2 years.* These changes apply to both statutory rape (Section 15, relating to penetrative consensual sexual acts) and statutory sexual violation (Section 16, relating to nonpenetrative consensual sexual acts).

The 2015 *SOA Amendment Act* has not changed the age of consent to sexual activity, which remains 16 years old – a fact that is pointed out in the preamble to the *Amendment Act*. The preamble also underlines the importance of ‘discouraging adolescents from prematurely engaging in consensual sexual conduct which may harm their development, and from engaging in sexual conduct in a manner that increases the likelihood of the risks associated with sexual conduct materialising’. Clearly, healthcare workers have a critical role to play in this regard.

### Bridging policy and practice

In practice, however, the judgement has not – and will not – substantially changed the complexities of service provision in practice until the amendments are made to the legal framework, and these changes are communicated to frontline service providers. We were therefore interested in better understanding how service provision happens in this context and in learning about healthcare providers’ strategies and experiences in providing SRH services to teenagers. We spoke to 28 healthcare workers across the Western Cape and have hosted a series of workshops with healthcare providers and stakeholders with experience in children’s law, public health, SRH rights, as well as representatives of local and national government. The results will be published elsewhere (Muller *et al*. 2016), and we will therefore only provide a short summary.

In general, we found that healthcare providers did not know what their obligations under the different Acts were. Many therefore rather explained to us ‘what we do here’. A particularly confusing area was the age of consent to sex, and the ages of consent to contraceptives and termination of pregnancies. Most healthcare providers were not aware that under the SOA, they were obliged to report consensual sex between 12–16 year olds to the police (under the provisions of the ‘old’ SOA that had not yet been overturned). Instead of referring to the police, many healthcare providers rather referred teenagers to a social worker. It became clear that nurses play complex and often contradictory roles when our results also clearly show that nurses play a complex and sometimes contradictory role when providing SRH services to adolescents. Whilst they are service providers who offer advice, support, counselling and care together with information about safe and healthy sexual behaviour on the one hand, they are also mandated to report knowledge of illegal sexual activity, and sexual abuse and violence, and thus act as law enforcers.

As a result of these conflicting duties, nurses struggled with confidentiality for their patients. In cases where the teenage patient was brought to the clinic by a family member, these conflicting roles are aggravated even more for nurses. Nurses, therefore, were caught between protecting confidentiality and acting in the best interests of the child. Nurses’ own values and attitudes (often as mothers themselves) were an important factor in understanding how they provide services to teens.

What emerged most clearly is that more guidance is needed for healthcare providers holding these competing roles and conflicting responsibilities. To this extent, we have summarised three hypothetical scenarios to provide an overview of the legislative framework and obligations, as well as recommendations coming from the service providers we worked with (see Table 4).

**TABLE 4:** Strategies for navigating conflicting laws.

Scenario	Acts and obligations	Recommendations
Girls (aged 12-16 years) seeking termination of pregnancy	**TOP Act:** No minimum age for a TOP, no parental consent needed. **SOA:** Children can only consent to sex from the age of 16. Sex with children under 12 must be reported to the police. All nonconsensual sexual activity with children (regardless of age) must be reported to police. Currently no provisions for consensual sex between children aged 12 to 15 years (pending legislative reform). ***Children’s Act:*** Age of consent to medical treatment is 12 years (with sufficient maturity).	Counsel the girl on her options in a nonjudgemental way. If you have doubts about the sexual partner or the voluntariness of the relationship, try to address these in the counselling session or refer to a social worker. Do not make this a condition for obtaining the TOP. If you have grounds to come to a conclusion of abuse, refer to a social worker or report to a designated child protection organisation or the police. Inform the girl of these steps. In accordance with the girl’s decision, refer for TOP or for antenatal care.
Teenagers (aged 12-16 years) seeking condoms, contraception or HIV testing services	***Constitution, National Heal**t**h Act:*** Everybody has the right to access health care, including sexual and reproductive health care. No parental consent is needed. ***Children’s Act*:** Children above 12 years of age may not be refused condoms and contraception. Provision must be kept confidential. ***SOA:*** Children can only consent to sex from the age of 16. Sex with children under 12 must be reported to the police. All nonconsensual sexual activity with children (regardless of age) must be reported to police. Currently no provisions for consensual sex between children aged 12 to 15 years (pending legislative reform).	Counsel teenagers on contraceptive options and safer sex in a nonjudgemental way. If you have doubts about the sexual partner or the voluntariness of the relationship, try to address these in the counselling session or refer to a social worker. Do not make this a condition for obtaining contraceptives. If you have grounds to come to a conclusion of abuse, refer to a social worker or report to a designated child protection organisation or the police. Inform the teenager of these steps.
Two male teenagers (aged 12-16 years) in a relationship seeking advice on safer sex	All of the above legislation applies equally to same-sex and opposite-sex relationships. ***Constitution:*** No discrimination based on sexual orientation (Bill of Rights 9[1]).	Educate yourself and your colleagues about same-sex practices and HIV/STI prevention. Ensure that your clinic stocks condoms for anal sex, lubricant and dental dams. Provide services in a nonjudgemental way. If you lack the knowledge to counsel these teenagers, refer to an LGBTI health service (see resources at the end of the article) and seek training for future consultations.

SOA, Sexual Offences Act; TOP, termination of pregnancy.

### Recommendations to improve teenage sexual and reproductive health service provision

Our research clearly shows that healthcare providers need to be better equipped to navigate the different obligations that they have under the existing legal, professional and ethical frameworks. We therefore have the following recommendations to improve adolescent reproductive health service provision.

### Better training of healthcare providers on the legal context in which they provide sexual and reproductive health services

One of the outcomes of our project are guidelines for all healthcare workers providing SRH services to adolescents, which we have developed in collaboration with the Department of Health. These will be available through Department of Health and through our Unit. Because the reality of short-staffed and over-burdened services often makes it impossible to send people to trainings, healthcare providers who want to skill themselves independently to understand the legal context and changes in the law can do so at their own pace and in their own time.

### Ongoing advocacy

Given that Sections 15 and 16 of the 2007 SOA have been amended, we need continued advocacy to ensure that the revised provisions are adequately disseminated to healthcare workers and other stakeholders. In addition, we need ongoing advocacy and tools to ensure that the changes in the law are translated into practice so that healthcare workers properly understand the reality of teenage sexuality and are able to provide adequate protection from sexual violence whilst at the same time not condemning consensual sexual exploration as part of a healthy developing sexuality. Updated plain-language guidelines for the provision of services to teenagers are an important part of these efforts.

We also believe that the voices of healthcare providers are critical to law reform and other policy process to ensure that the frameworks that govern service provision take into account the context in which health services are offered and understand the potential roles that healthcare providers will have to play in the enforcement of legislation.

### Websites for further information

1. The Gender, Health and Justice Research Unit (research report and training material for healthcare providers, www.ghjru.uct.ac.za).
2. IBIS Reproductive Health (advice and support for healthcare providers, www.ibisreproductivehealth.org).
3. UCT Children’s Institute: The Children’s Act Guide for Health Professionals (available at www.ci.uct.ac.za).
4. Triangle Project (support, advice and SRH services for lesbian, gay, bisexual, transgender and intersex people, www.triangle.org.za).
5. Gender Dynamix (support and advice for transgender people and guidelines for healthcare providers, www.genderdynamix.org.za).
6. Health4Men (Support, advice and SRH services for gay men and men who have sex with men, www.health4men.co.za).

### Clarifications of terms

1. Under certain conditions, women and girls can request a TOP beyond the 12-week gestation period (up to 20 weeks), for instance if the pregnancy is the result of a rape, if the foetus is at risk for severe mental or physical abnormity or if there is a substantial risk for the physical or mental health of the pregnant woman or girl. Whereas abortions in later trimesters can only be carried out by medical doctors, first trimester abortions can be carried out by doctors as well as sufficiently trained midwives and nurses. Abortions are available at healthcare facilities that are equipped in terms of staffing, equipment and facilities and that have been designated by the Minister of Health. Since an amendment of the law in 2008, abortions can also be carried out at facilities that have not been designated as long as they are sufficiently equipped and have a 24-hour maternity service.
2. A patient’s right to confidentiality under the NHA may be limited where a law allows or requires the disclosure of his or her medical information. This is different from the CTPA, which does not provide for limitations of patient confidentiality.
3. This standard is set out in Section 9 of the *Children’s Act*. Section 7 of the Act sets out the factors that should be considered when making a determination of the best interests of the child and includes considerations such as the nature of the relationship between the child and their parents or care-giver; the child’s age, maturity and stage of development; gender; background; physical and emotional security; intellectual, emotional, social and cultural development; and other relevant characteristics.
4. The Act stipulates that ‘contraceptives other than condoms may be provided to a child on request by the child and without the consent of the parent or care-giver of the child if the child is at least 12 years of age’, on the condition that the child received medical advice and a medical examination to ensure there are no medical reasons not to provide the contraceptives. Aside from these physical health precautions, there is no requirement that the child undergo any further counselling before being issued with the contraceptives. Section 134 of the *Children’s Act* sets out stringent penalties of a fine and/or imprisonment for up to 10 years for anyone who refuses to provide contraceptives within this framework.
5. ‘Abuse’ is defined in the *Children’s Act* as ‘any form of harm or ill-treatment deliberately inflicted on a child’ and includes assault, bullying, exploitation, behaviour that may psychologically or emotionally harm the child and sexual abuse.
6. The 2007 SOA also created a defence for the offence of statutory sexual assault. Accordingly, it was a valid defence to a charge of statutory sexual assault if both accused persons were children at the time of the offence, and the age difference between them was not more than 2 years at the time of the offence. Thus, if a 14–year-old and a 12-year-old consensually kissed under the 2007 SOA, the accused could use the defence provided by s 54(1)(b) of the SOA. This defence, however, only applied to nonpenetrative forms of sexual conduct and did not, therefore, provide a defence for statutory rape.
  This so-called ‘close-in-age’ defence has essentially been incorporated into the Act under amendments brought about by the 2015 *SOA Amendment Act* by decriminalising consensual sexual activity (both penetrative and nonpenetrative) where both parties are 12–15 years old, regardless of the age difference between them, or where one party is between 12 and 15 years old and the other between 16 and 17 years old and the age gap between them is not more than 2 years.
7. This provision is broadly drafted and applies to all sexual offences against children, including rape, sexual assault, child pornography, sexual exploitation and sexual grooming. Thus, any person who knows that a child has been raped or sexually assaulted must report this knowledge to the police. The reporting obligation also applies to consensual sexual acts between teenagers that constitute statutory rape or statutory sexual assault under the SOA.
